# Adjustable Pediatric Prosthetic Sockets to Accommodate Growth in Children and Adolescents With Limb Loss

**DOI:** 10.1016/j.arrct.2026.100670

**Published:** 2026-06-26

**Authors:** Timothy R. Dillingham, Jessica Kenia, Laura Prosser, James Marschalek, Frances Shofer, Liliana E. Pezzin

**Affiliations:** a Department of Physical Medicine and Rehabilitation, University of Pennsylvania Perelman School of Medicine, Philadelphia, PA; b Division of Rehabilitation Medicine, Children’s Hospital of Philadelphia, Philadelphia, PA; c Department of Pediatrics, University of Pennsylvania Perelman School of Medicine, Philadelphia, PA; d Engineering Department, Advanced Design Concepts, Oconomowoc, WI; e Department of Emergency Medicine, University of Pennsylvania Perelman School of Medicine, Philadelphia, PA; f Division of Health Policy, Economics and Data Analytics, Institute for Health and Humanity, Medical College of Wisconsin, Milwaukee, WI

**Keywords:** Amputation, Limb deficiency, Pediatric, Prosthesis, Rehabilitation

## Abstract

**Objective:**

To evaluate the feasibility, comfort, user satisfaction, and functional performance of an adjustable, immediate-fit prosthetic socket in children and adolescents with lower-limb loss.

**Design:**

Prospective clinical trial.

**Setting:**

Gait and biomechanics lab in an outpatient rehabilitation hospital.

**Participants:**

Twenty-eight pediatric participants (14 boys, 14 girls; average age 11.5±4.0y) were enrolled; 26 were eligible for the home trial.

**Intervention:**

Each participant was fit with an adjustable, immediate-fit prosthesis, which was compared with the users’ conventional devices.

**Main Outcome Measures:**

Prosthetic Evaluation Questionnaire (PEQ) scores (use and satisfaction) with additional items on comfort; temporal gait parameters (gait speed, single-limb support); intrasocket pressure measurements; and adverse events (falls, skin irritation, device issues).

**Results:**

Fifteen participants completed follow-up. Overall PEQ scores favored the adjustable device, although not significantly (45.8 vs 42.5, *P*=.29). Ease of socket adjustment demonstrated a significant advantage for the adjustable prosthesis (mean difference=1.53, *P*=.0019). Participants also reported improved temperature and perspiration control (mean difference=1.07, *P*=.0007). Walking speed did not differ between devices (*P*=.24), and intrasocket pressures were comparable (*P*=.74). No skin irritation, skin breakdown, or mechanical failures were observed or reported.

**Conclusions:**

The adjustable, immediate-fit pediatric prosthetic system was feasible and well accepted. It performed comparably with conventional devices on gait and pressure metrics and demonstrated ease of use and improvements in factors associated with skin integrity. These findings suggest that adjustable sockets may offer a practical, affordable alternative for children and families seeking a device that can accommodate growth.

Children with limb loss or limb deficiencies constitute a unique group of prosthetic users whose rapid growth often causes them to outgrow traditional sockets quickly. High levels of activity further require prosthetic systems that are comfortable, durable, and adaptable enough to support full participation in daily, recreational, and sports activities. Engagement in such activities is well documented to improve health, psychological well-being, and social integration.^1^, ^2^, ^3^

Despite advances in prosthetic componentry, fabrication techniques have remained largely unchanged.^4^ The process is time- and labor-intensive, involving casting, molding, and iterative modifications to achieve an acceptable fit.^5^ Final fabrication typically requires several weeks and results in a rigid socket that cannot accommodate limb changes as the child grows.^6^, ^7^, ^8^, ^9^, ^10^ Consequently, the ongoing cycle of obtaining new conventional prostheses imposes substantial time, logistical, and financial burden on both children and their parents. Pediatric patients and their families spend an average of 42.7 hours a year on prosthetic care, including travel and appointments, with school-age children missing approximately 5 days a year of school.^11^ Extended leave of absence for reasons related to limb loss was reported by 52% of dual-parent mothers, 24% of dual-parent fathers, and 69% of single parents.^11^ Adolescents typically require prosthetic follow-up every 4-6 months because of growth-related limb changes.^12^

Because of ongoing growth and physical development, children with lower-limb amputations require frequent prosthetic replacement, with estimates ranging from 6 to 22 prostheses before reaching skeletal maturity.^13^^,^^14^ Individual prostheses may cost approximately $3,000-$15,000 depending on amputation level and device configuration, and the cumulative economic burden is substantial.^13^^,^^14^ Lifetime prosthetic expenditures, including replacement devices, maintenance, repairs, and component replacement, have been estimated at $73,000-$116,000 by age 18.^13^^,^^15^ Annual coinsurance and copays for prosthetic services account for nearly half of the overall annual out-of-pocket expenses for these families.^15^ Insurance coverage is often inconsistent, with 28% of privately insured families reporting claim denials.^11^ Secondary or sport prostheses coverage is very limited and often deemed not medically necessary.^11^

Comfort and skin integrity are critical to successful prosthetic use, yet discomfort remains a persistent challenge.^16^, ^17^, ^18^, ^19^ In a survey of 258 pediatric prosthetic users, skin breakdown on the residual limb was a common problem reported by 74% of children using lower-limb prostheses, resulting in 16% of the children temporarily discontinuing use of their prostheses.^20^ Twenty-four percent reported periods of prosthesis nonuse because of pain in the limb from the prosthesis.^20^ Serious perspiration problems were reported in 20% of respondents and were cited as a factor limiting prosthetic use.^20^ These findings highlight persistent challenges in current prosthetic care and underscore the limitations of conventional prosthetic technology for this highly active population.

To address these challenges, an adjustable prosthesis (iFIT Prosthetics, LLC)^a^ was developed for rapid fitting. In contrast to conventional fabrication, these prostheses are fit in one 2–3-hour session using hand tools. The socket is made from advanced injection-molded materials and is mass-produced with consistent quality, providing a cost-effective option. As off-the-shelf devices require fewer components and fabrication steps, they may reduce overall costs. Each device is thoroughly tested to ensure strength, durability, and conformity to our adapted International Organization for Standardization for prosthetic components.^21^ The lightweight design is comparable to conventional pediatric sockets and can support weights up to 120 lb/54 kg, whereas the adult socket, used for teens, can support up to 260 lb/118 kg. Devices are customized through trimming, heat molding, and internal padding to optimize fit and comfort.

For this study, we evaluated adjustable transfemoral, transtibial, and Syme’s sockets in children and adolescents. These devices all feature an adjustable base cup that can expand to accommodate a range of limb diameters and are total surface bearing in design (fig 1). They are padded with a 5 mm neoprene liner, and an optional kit with extra pads is provided. A silicone liner, 3 mm is recommended, and pin-suspension system is used to suspend the prosthesis on the limb.^22^ The adjustable transtibial and Symes devices have overlapping flaps and an internal pulley mechanism that allows the user to adjust the tightness of the socket (fig 2). Once the prosthesis is sufficiently tightened, the adjustment cord is wrapped around a cleat located on the upper lateral part of the socket. The transtibial devices come precut to different lengths or can be customized for children with longer limbs or Symes amputations. The Symes amputation is accommodated by lower-profile feet and componentry (fig 3).

**Fig 1:**
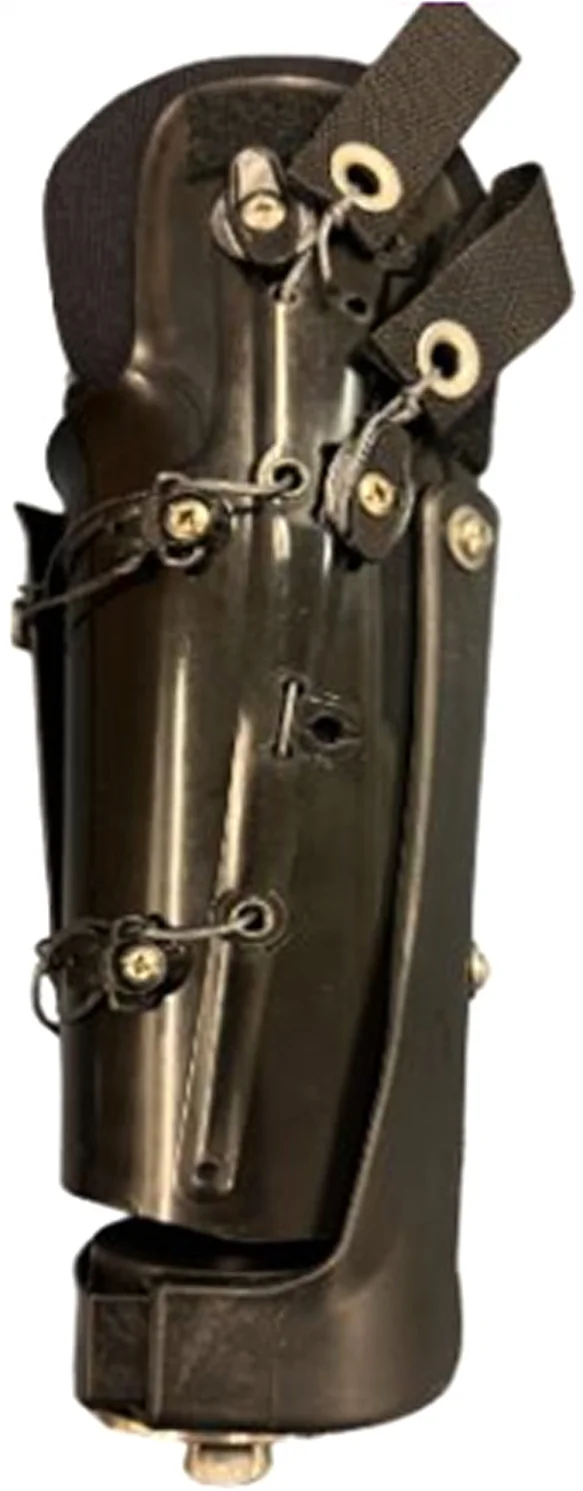
The transtibial prosthesis with pull cord adjustment system and adjustable bottom cup.

**Fig 2:**
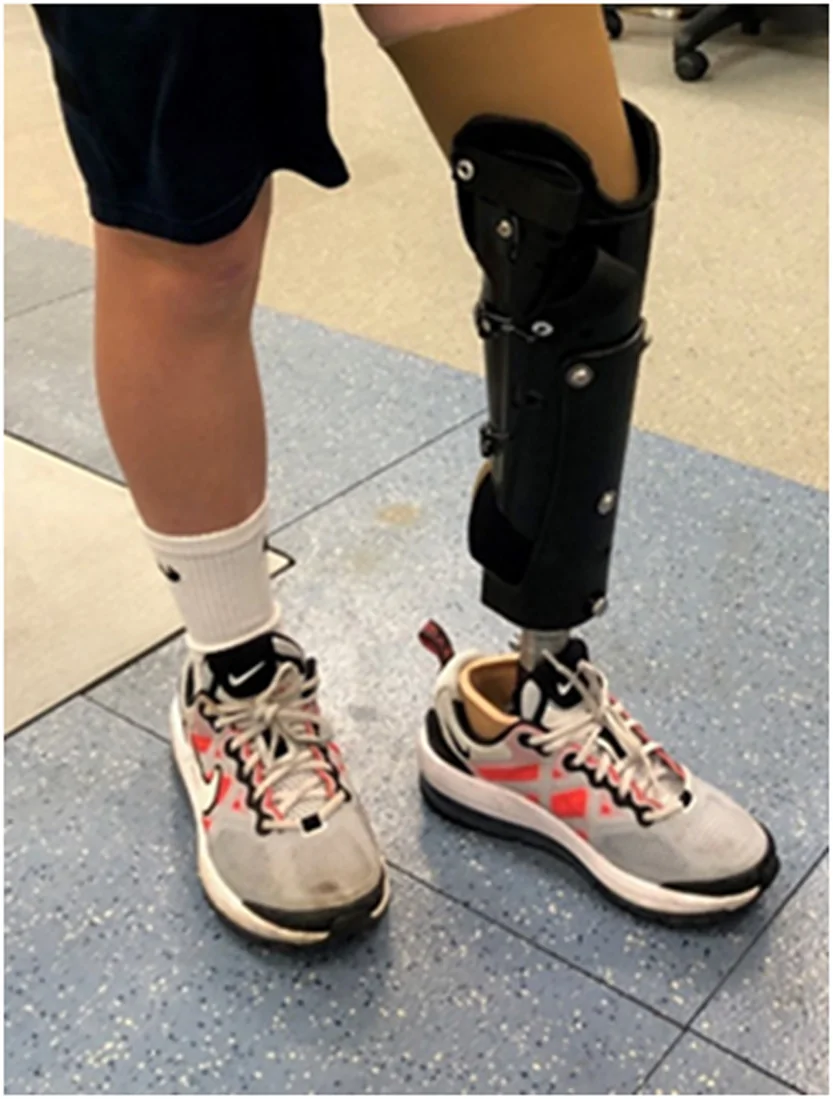
Transtibial device on a study patient.

**Fig 3:**
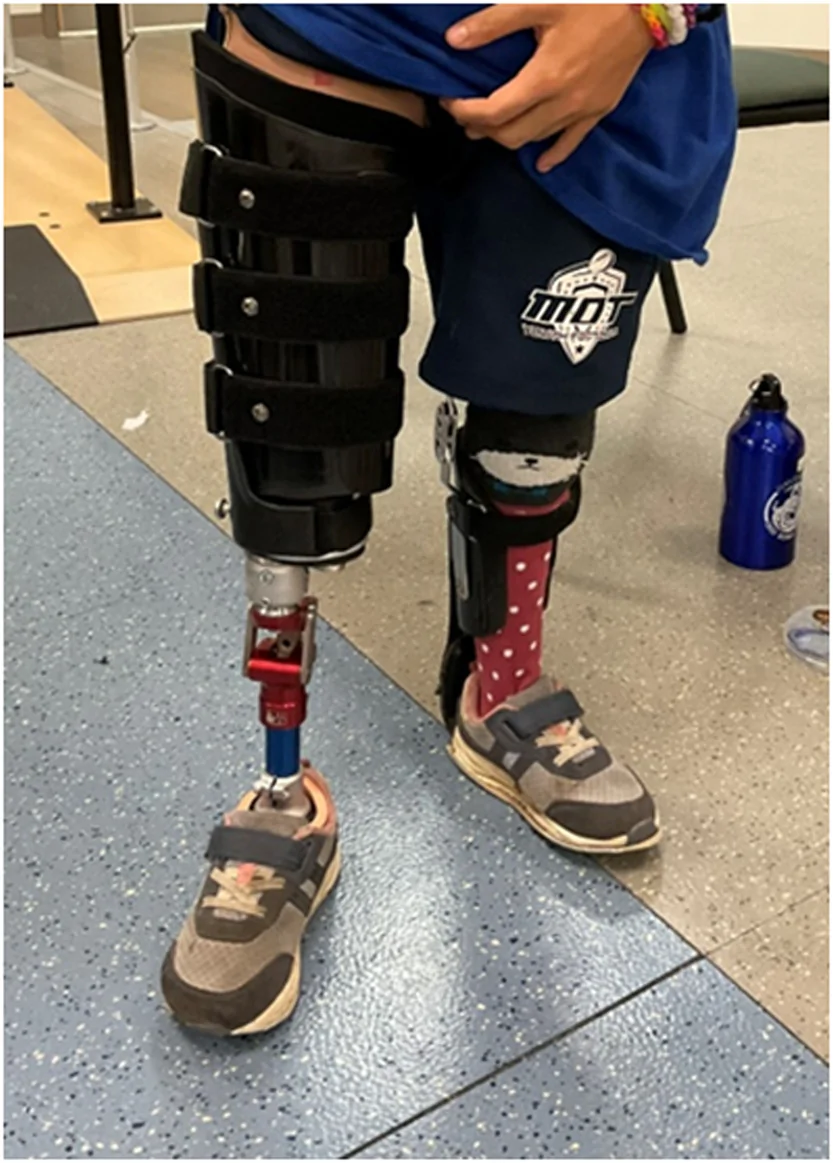
Transfemoral device on a study patient.

The transfemoral prosthesis also features two overlapping posterior flaps that wrap around the residual limb and are closed by the user through adjustable Velcro straps (fig 4). This design incorporates a subischial brim. Prior biomechanical and user-centered evaluations on adults have demonstrated that subischial brims confer superior comfort, improved hip range of motion, and enhanced functional mobility compared with traditional ischial-containment designs.^23^ The transfemoral socket also employs a pin-suspension liner and locking mechanism. These systems work with commercially available feet, pylons, suspension, and liners.

**Fig 4:**
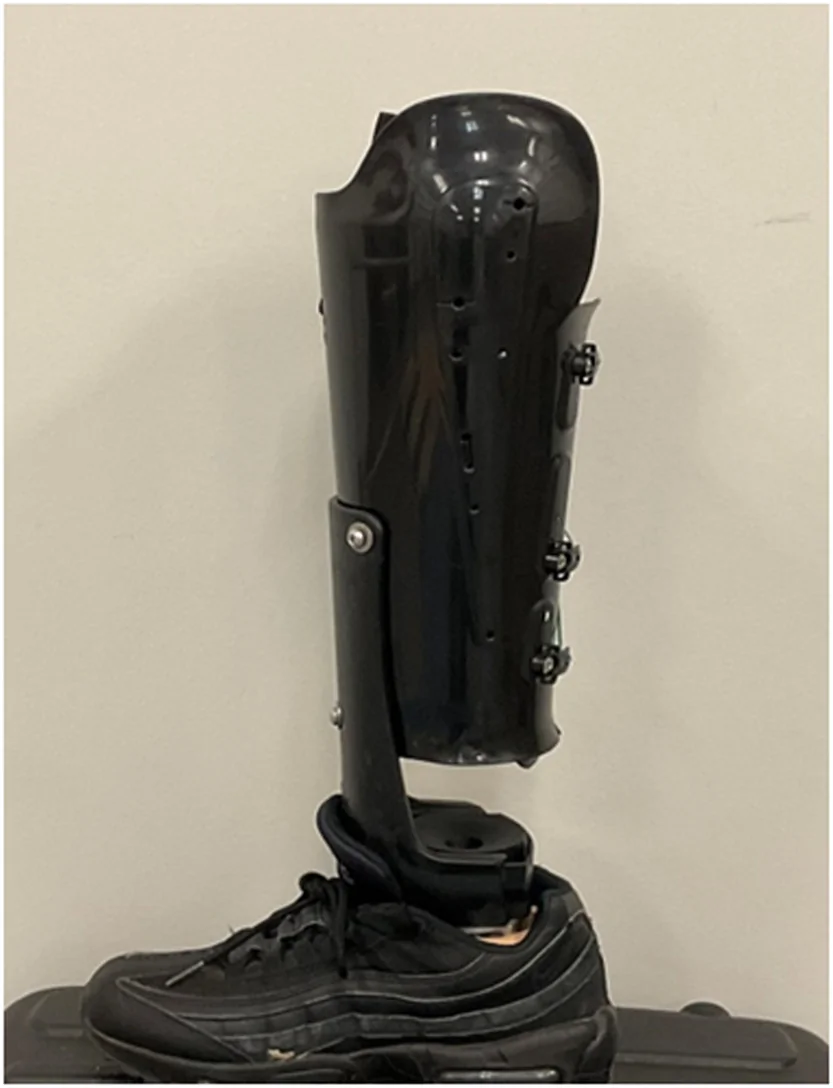
The Symes adjustable prosthesis features 2 overlapping posterior flaps that wrap around the residual limb and are closed by the user. The bottom area is open to accommodate the bulbous end. A pin-suspension system is used.

The objective of this study was to evaluate the feasibility, functional performance, and user experience of the adjustable, immediate-fit socket compared with each child’s existing conventional prosthetic device.

## Methods

Participants aged 3-18 years old were recruited from local hospitals, including the University of Pennsylvania and Children’s Hospital of Philadelphia, over a 2-year time period from 2023 to 2025. We also posted a recruitment advertisement on the Amputee Coalition of America website. The families of potential participants were encouraged to contact the research coordinator for screening before participation. Eligible participants were required to be at least 4 months postamputation,^24^ ambulating with a functional prosthesis with or without an assistive device. Children with limb loss from any cause—trauma, congenital limb deficiency, or malignancy—were eligible, provided they had intact protective sensation in the residual limb as assessed by the Physiatrist Principal Investigator during the initial evaluation. Exclusion criteria included: (1) open skin lesions; (2) significant residual limb pain, including phantom pain or neuroma; and (3) neurological disorders (eg, stroke, spina bifida, severe polyneuropathy) resulting in contralateral weakness or substantial gait impairment. The University of Philadelphia served as the Institutional Review Board of record for the approval of all research procedures (IRB #849980). All parents or legal representatives provided written informed consent before the start of any study activities. Written assent of subjects was obtained for all participants 7 years of age and older. All outcome measures were collected by the same Research Coordinator. The Principal Investigator, who developed and designed the prosthesis, also performed all fittings, modifications, and alignment of the devices. The PI was not present for any data collection, nor did he participate in statistical analysis.

During the initial visit, participants completed a baseline survey based on their conventional prosthesis using the Prosthetic Evaluation Questionnaire (PEQ),^25^ which assesses use and satisfaction with their prosthetic device. Parents or caregivers assisted younger children by reading the questions aloud and recording the children’s responses. Questions were also added assessing skin breakdown (open wounds) and falls in the past 3 months. Each child had an intrasocket pressure analysis measured using Fujifilm^b^, which is a thin, flexible sheet that can be cut and placed into key areas of the sockets. The Fujifilm was cut into 1” squares and placed on the participant’s liner for the measurement of maximum pressure profiles in the transtibial socket over the anterior tibia, distal tibia, medial tibial flare, fibular head area, and gastrocnemius areas for the transtibial sockets. For the transfemoral sockets, these areas were: end of femur, posterior thigh, anterior thigh, lateral thigh, and medial thigh locations. Once the Fujifilm was in place, the subjects donned their socket and ambulated for 2 minutes, after which the paper was removed and promptly scored.

Walking gait speed was measured using a Vicon motion analysis system. Each participant was instructed to walk at a self-selected, comfortable pace along a 10-meter laboratory walkway. Six walking trials were completed, and at least 4 valid trials were used for gait speed analysis. We initially distributed pedometers to obtain baseline activity levels, placing them directly on the prosthesis to ensure accurate step capture. However, because of the children’s high activity levels and the resulting frequent device breakage, the pedometer component of the protocol was discontinued because it did not yield adequate usable data.

Once the assessments were completed, participants had their limb length and circumferences measured. The appropriate socket size was chosen based on their measurements, along with a prefabricated 3 mm silicone locking liner and a prosthetic dynamic-response foot corresponding to their shoe size. The feet used were the WillowWood^c^ Pediatric Impulse foot for smaller sizes (18-22 cm) and the College Park^d^ Celsus foot for larger sizes (23-27 cm). Children with transfemoral limb loss were fit with a mechanical 4-bar linkage knee. We attempted to match the foot and knee components to those the child normally used; however, in many cases, this was not possible because some children’s existing componentry exceeded the study’s budgetary limits. For the fitting, each participant wore a silicone locking liner and had the prosthesis fit directly to their residual limb. A pylon and foot were attached to the correct height, and the prosthesis was aligned.

Modifications were made to the sockets to accommodate each limb. These modifications included heat molding the brim or posterior socket to prevent rubbing, padding the neoprene liner to alleviate pressure over bony prominences, and creating distal posterior socket cutouts for children with a Syme’s amputation and a bulbous residual limb. Once a participant was fit, they ambulated for an extended period in the lab to ensure no other modifications were needed. Participants were instructed to wear the adjustable prosthesis as much as they wished and were permitted to use their conventional prosthesis if any issues arose. They returned for a follow-up visit two months after the initial fitting to repeat the outcome measures on the adjustable prosthesis. Because of busy schedules for children and parents, this was a target date. Two months was the minimum timeframe for the trial, and they were generally scheduled within a week of the target date.

To characterize the study population, we calculated frequencies and percentages for categorical variables and means and standard deviations for continuous variables. Data from transtibial, transfemoral, and Syme amputations were combined to allow for an overall comparison between the adjustable test socket and each participant’s conventional prosthesis. To compare responses to individual PEQ items and overall patient satisfaction between the adjustable device and each participant’s conventional device, we used paired *t-*tests. Mean differences were approximately normally distributed, showing only mild left skew. Results are reported as mean differences with corresponding 95% confidence intervals between the adjustable and conventional sockets. All analyses were performed using SAS statistical software (version 9.4; SAS Institute)^e^. Figures were created using GraphPad Prism (version 10.6.0; GraphPad Software, Inc)^f^.

## Results

A total of 28 pediatric participants (14 boys, 14 girls; mean age 11.5±4.0y; range 4-18y) enrolled in the study. Of these, 26 participants took the prosthesis home, whereas two younger children (ages 5 and 7) had difficulty following instructions during the initial fitting and were not provided with the device. Fifteen participants returned for follow-up (tables 1 and 2). Attrition was attributable to factors such as travel distance, medical issues, school commitments, and challenges in scheduling follow-up visits.

**Table 1:** Summary statistics

	Completed (N=15)	%	Lost to Follow-Up (N=11)	%	*P* Value
Sex					>.99
Men	7	46.7	5	45.4	
Women	8	53.3	6	55.6	
Level of amputation					.78
Transtibial	6	40.0	3	27.3	
Symes	3	20.0	3	27.3	
Transfemoral	6	40.0	5	45.5	
Etiology					.037
Congenital	10	66.7	5	63.6	
Trauma	2	20.0	2	18.2	
Cancer	0	0	4	36.3	
Other	3	13.3	0	0	
Conventional suspension					.85
Anatomical	5	33.3	4	36.3	
Pin	4	26.7	4	36.3	
Suction	4	26.7	2	18.2	
Lanyard	2	6.7	1	9.1	
Hours/day of wear (conventional prosthesis)					.097
1-3	0		1	9.1	
4-7	2	13.3	1	9.1	
7-9	2	13.3	5	45.5	
9+	11	73.3	4	36.4	
Hours/day of wear (adjustable prosthesis)					–
1-3	11	73.3			
4-7	1	6.7			
7-9	1	6.7			
9+	2	13.3			

NOTE. *P* values were calculated using Fisher’s Exact test.

**Table 2:** Characteristics of participants who completed the study

Participant	Age (Y)	Gender	Type	Etiology	Length of Time Using a Prosthesis	Conventional Suspension	Foot Type
1	17	Men	Transtibial	Congenital	15y	Suction	Ossur Pro-Flex
2	9	Men	Symes	Congenital	8y	Anatomical	Dynamic response
3	16	Women	Transtibial	Other: infection complication	7mo	Pin	Ossur Flex foot
4	9	Women	Knee disarticulation	Trauma	6y	Pin	Dynamic response
5	10	Men	Transtibial	Congenital	10y	Anatomical	Fillauer pediatric blade
6	19	Women	Transfemoral	Other: vasculitis	7mo	Suction	Ossur Flex foot
7	17	Men	Transtibial	Trauma	8y	Suction	Ossur Pro-Flex w/ torsion
8	15	Women	Transfemoral	Congenital	12y	Lanyard	Dynamic response
9	12	Women	Symes	Congenital	4y	Anatomical	Sach
10	12	Women	Transtibial	Other: arteriovenous malformation	1y	Pin	Ossur Flex foot
11	11	Men	Transtibial	Congenital	8y	Suction	Ossur Flex foot
12	11	Men	Transfemoral	Congenital	7y	Pin	Ossur Flex foot
13	12	Women	Transfemoral	Congenital	7y	Anatomical	Ossur Flex foot
14	4	Women	Transfemoral	Congenital	3y	Lanyard	Sach
15	12	Men	Symes	Congenital	10y	Anatomical	Ossur Flex foot

Table 1 shows the summary statistics for the children and adolescents who enrolled in the study. Baseline characteristics were generally similar between participants who completed the study and those lost to follow-up. Sex distribution did not differ between groups (46.7% vs 45.4% men; *P*>.99), and the level of amputation was comparable across categories (*P*=.78). The conventional prosthesis suspension type was also similar between groups (*P*=.85). Although congenital limb loss was the predominant etiology in both groups (66.7% vs 63.6%), the overall distribution of etiologies differed significantly (*P*=.037), with cancer-related amputations occurring only among those lost to follow-up (20%) and “other” causes reported only among completers (13.3%). Daily wear time of the conventional prosthesis trended toward higher usage in the completed group (73.3% reporting ≥9 h/d vs 36.4% among those lost to follow-up), although the difference did not reach statistical significance.

**Fig 5:**
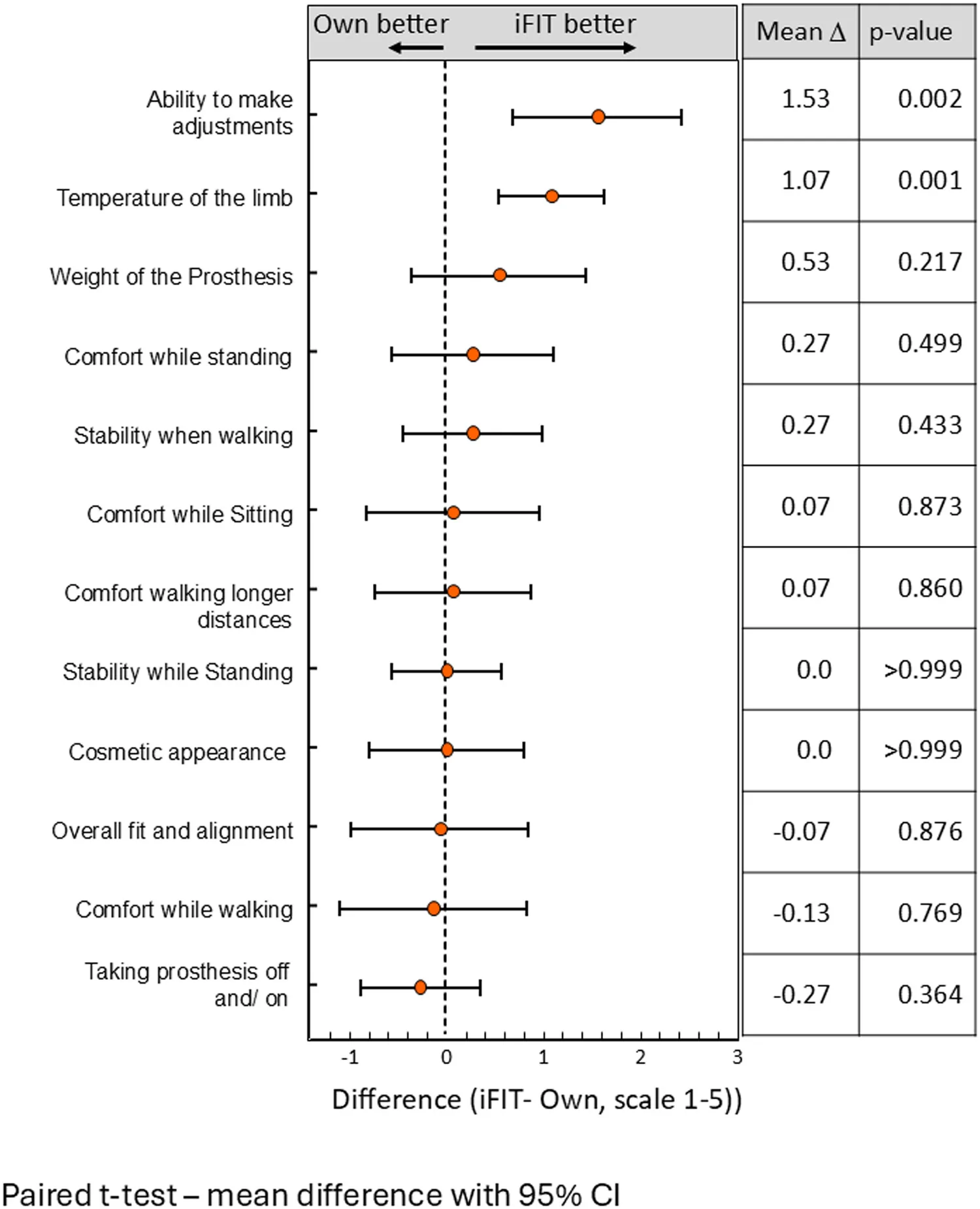
Paired *t* test analysis of PEQ scores comparing the adjustable prosthesis with participants’ conventional devices.

**Fig 6:**
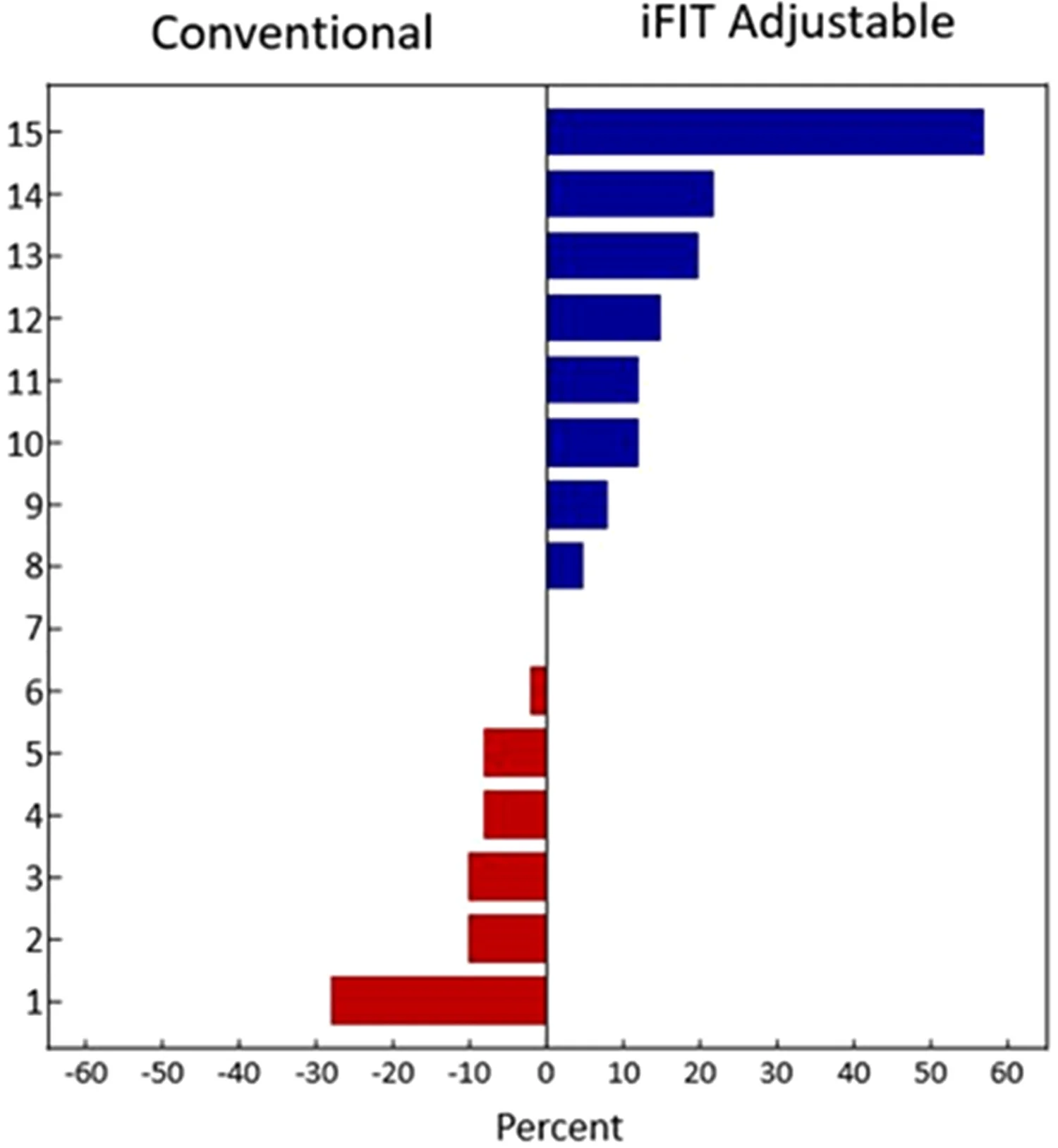
Percent change in overall PEQ score from each participant’s conventional prosthesis to the adjustable device. Results are arranged from those preferring the adjustable prosthesis to those who preferred their conventional device.

Compared with participants’ conventional prostheses, the PEQ demonstrated significantly higher scores for the adjustable device in adjustability (mean difference=1.53, *P*=.0019) and in temperature regulation, reflected by reduced sweating (mean difference=1.07, *P*=.0007) (fig 5). Overall satisfaction, assessed using the Pediatric PEQ, was higher for the adjustable device than for the conventional prosthesis (45.8 vs 42.5), although this difference did not reach statistical significance (*P*=.29). Preferences were evenly divided: half of the participants reported favoring the adjustable prosthesis (n=8), and the other half preferred their conventional device (n=7). One participant expressed no preference between the two (fig 6).

There were no significant differences in gait speed between devices. Mean walking speed using the conventional prosthesis was 1.04±0.20 mph compared with 0.98±0.27 mph for the adjustable device (mean difference=0.065 mph; *P*=.24). This is similar to the average walking speed reported in a past study, which was found to be 1.23 (±0.20) m/s with a range of 0.99-1.61 m/s for children with varying lower-limb amputation.^26^ On the basis of the pediatric gait literature, children who ambulate with a prosthesis typically walk at slower self-selected speeds than their developing peers.^26^

**Fig 7:**
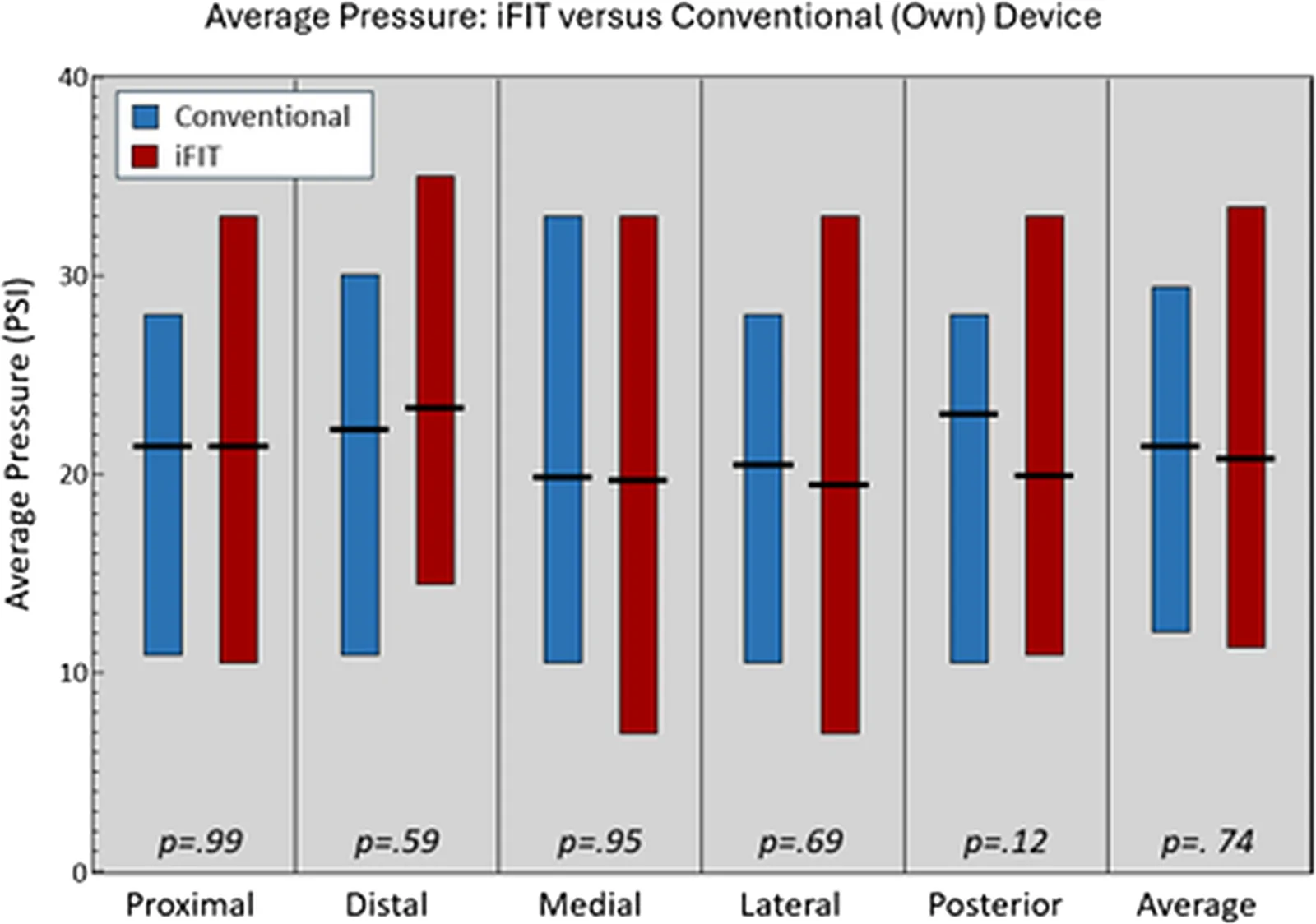
Comparison of intrasocket pressure profiles between the adjustable and conventional sockets using Fujifilm Prescale Extreme Low*.*

Similarly, there were no statistically significant differences between devices in intrasocket pressure, either overall or at individual measurement sites. The mean peak pressure for conventional sockets was 21.36±5.48 psi, compared with 20.74±6.58 psi for the adjustable device (mean difference: 0.62psi; *P*=.74), as illustrated in figure 7.

Before enrollment, 8 participants (31%) reported episodes of skin breakdown associated with their conventional prosthetic sockets within the preceding 3 months. During the study period, no participants reported new skin breakdown while using the adjustable prosthesis, and no skin integrity issues were observed at follow-up examination. Three children reported falls while using the adjustable prosthesis. Two of these occurred in participants wearing the adjustable transfemoral prosthesis who also reported falls when using their conventional devices. The remaining fall occurred in a participant using the adjustable transtibial prosthesis; the child attributed the incident to jumping out of a car without his usual shock-absorbing flexible foot on his conventional prosthesis. None of the falls resulted in injury. No socket structural failures occurred during the trial. One participant experienced a broken cleat, but she was able to securely don and wear the prosthesis by looping both tightening cords around the remaining cleat.

When asked whether they would recommend the adjustable prosthesis to other children, 93% of participants responded affirmatively. The cohort was generally active: 73% reported participating in running or sports activities with their conventional prosthesis. With the adjustable prosthesis, most participants (86.7%) reported that walking was their primary activity, whereas 53.3% indicated they were unable to run or participate in sports while using it. We speculate that limitations in the foot component, rather than the socket design, were the primary constraint on running for many participants. By comparison, 39% of participants also reported difficulty running with their conventional prosthesis.

Three of the participants transitioned from wearing their conventional prosthesis to wearing the adjustable prosthesis as their main prosthesis (participants 6, 19, and 16). Two of these individuals (participants 6 and 16), both with transtibial limb loss that had occurred between 1 year and 8 months prior and had previously used pin-lock systems, continued full-time use for approximately 18 months to date. Among the remaining participants, most reported using the adjustable prosthesis along with their conventional device or as a backup. Only 1 participant returned the prosthesis after completing the 2-month trial period, stating she liked her existing transfemoral socket.

## Discussion

Our findings support the short-term feasibility of the adjustable prosthesis as a daily wear option for children and adolescents. According to self-reported outcome measures, the adjustable sockets performed at least as well as conventional devices in enabling safe ambulation without compromising gait speed or functional mobility during community ambulation or climbing stairs. Half of the participants preferred the adjustable socket to their current traditionally fabricated device. These findings were echoed in a recent meta-analysis, which reported that adjustable sockets are generally perceived as more comfortable in adults in comparison with traditional rigid sockets.^27^

A particularly relevant result was the absence of skin breakdown or irritation while using the adjustable socket during the two-month trial period. Eight participants reported experiencing skin breakdown or irritation during the 3 months before enrollment while using their conventional prostheses. Prior research indicates that skin breakdown affects up to 74% of pediatric users and is a major contributor to reduced prosthesis use.^18^ Although the trial duration and daily wear time were limited, the lack of skin-related issues with the adjustable socket is likely attributable to its customizable fit, total surface bearing design, and socket neoprene liner (transtibial sockets) with optional supplementary padding for high-risk areas such as the distal tibia.

Another notable finding was the participants’ perception of better temperature regulation when using the adjustable device. This aligns with previous adult studies involving similar adjustable systems.^28^ Although all participants wore silicone liners that limited direct airflow to the skin, the adjustable socket design may have influenced the thermal environment within the prosthetic system through increased air exchange around the liner, and reduced heat retention compared with a fully enclosed rigid socket. Because temperature and perspiration were not directly measured, the mechanism underlying these perceived improvements remains uncertain. Most participants reported fewer hours of prosthesis wear during the study, which could contribute to the perception of less sweating.

The absence of differences in gait speed between the adjustable and conventional devices further indicates that the adjustable prosthesis does not adversely affect gait.^29^ Comparable findings have also been reported in adult users of immediate-fit adjustable sockets.^30^ The absence of a decline in gait speed suggests that the adjustable design can maintain functional mobility despite its increased adjustability, even in the presence of variability in prosthetic foot designs across participants.

The Fujifilm pressure analysis, although less precise for this purpose, provided useful comparisons of maximum sustained intrasocket pressures. These pressures represent the maximum sustained pressures in a particular area on the residual limb. Similar pressure profiles between devices suggest that children did not overtighten the adjustable socket and that pressure distribution is comparable with that of conventional sockets.

Most children experienced no difficulty putting on, taking off, or adjusting the adjustable prosthesis. Only two participants required parental assistance, but they similarly needed help with their conventional devices. Many children expressed enthusiasm for the internal pulley mechanism used to close the adjustable socket and appreciated being able to operate it independently. Consistent with these observations, ease of putting on and off did not differ significantly between devices, and no participants reported difficulties, even among those who previously used anatomically suspended sockets that did not require a lock or suspension sleeve in their traditional devices.

Several challenges arose during fitting. Younger children often had limited tolerance for the single-session protocol (∼3h for questionnaires, testing, and fitting), which was used to reduce family travel burden. Some were also hesitant to try a device that felt unfamiliar compared with their conventional socket. Only children willing to trial the prosthesis were sent home with it; two were not. Running was also limited in the adjustable socket because of the use of lower-impact feet that may be restrictive for running. Many participants participated in sports or recreational activities involving running and would need to switch to their conventional prosthesis with a higher-impact foot during these times.

Many of the children also had bony prominences at the end of their residual limbs, especially in cases with Symes amputations. Their conventional sockets were generally shaped to capture the distal flare of the limb and featured flexible inner liners to help don the socket. These bony areas or bulbous ends of the residual limb could be accommodated by cutting out parts of the socket that put pressure on them or by padding the area. Two children had shifting patellas leading to knee instability, one had a socket that lacked supracondylar support, and the other was accommodated with a thigh lacer with side joints. Both (Participants 3 and 15) were fit in a way to add extra supracondylar support to the knee area.

### Study limitations

An important limitation of this study is that the conventional prostheses did not use identical components to the adjustable devices. Although components—particularly feet and knees—were selected to match each participant’s ambulatory level, some were unavailable or exceeded the study budget. These differences may have affected outcomes independently of socket design. Future studies should use the same knee and foot components as those in participants’ conventional prostheses. All participants used prefabricated prosthetic liners, including those with atypically shaped residual limbs for whom custom-fabricated liners might ordinarily be prescribed.

Also, because of the small sample size of transtibial, transfemoral, and Symes participants, the data were combined to allow for a general comparison between sockets. As a result, the findings should be interpreted as representing the overall study population rather than any specific amputation level. Potential differences in fit, comfort, or function related to amputation level could not be evaluated and warrant investigation in larger studies.

Another limitation was that long-term tracking of the participants and inspection of the sockets were difficult because of travel and the need for families to take time off from work and school. As a result, some longer-term issues related to durability, fit, or skin health may not have been fully captured during the study period. It could also not be fully assessed whether the prosthesis could capture all longitudinal growth. However, the sockets studied are forgiving for varied lengths and will generally fit a range of 3-4 cm in length (ie, lengths 16-19 cm will fit a pediatric medium).

Selection bias may also be present, as participation was voluntary and families who chose to enroll may have been more motivated to try new prosthetic technologies or more dissatisfied with their existing devices. In addition, patient-reported outcome measures were completed either by the child or by a parent depending on the participant’s age and ability to respond. Differences in perspective between children and parents may have influenced responses.

## Conclusions

Our findings suggest that adjustable prostheses may be a viable option for pediatric users, whether worn full time or as a secondary device. The key design features of the prosthesis—adjustability and immediate fit—may offer potential to improve accessibility and availability of prosthetic services for some patients. This prosthesis is designed to have a longer prosthesis lifespan compared with conventional devices by way of accommodating growth, potentially offering greater convenience for patients and less time spent by parents in accessing prosthetic care. Finally, the comparatively low cost of the adjustable sockets makes them an attractive alternative for families seeking an affordable primary prosthesis or an additional device for activities such as water use. A larger multicenter, comparative effectiveness study with longer-term observation is needed to more fully evaluate the potential of adjustable prosthetic devices for this population.

## Suppliers

a. iFIT Prosthetics–transtibial, Symes, and transfemoral prostheses.
b. Fujifilm–prescale.
c. WillowWood–Impulse Pediatric Foot.
d. College Park–Celsus Foot.
e. SAS statistical software, version 9.4; SAS Institute.
f. GraphPad Prism, version 10.6.0; GraphPad Software, Inc.

## Disclosures

T.R.D. founded the company iFIT Prosthetics, LLC and is the major owner and director. He has a financial interest in the prosthetic system being presented in this article. He signed a National Institutes of Health compliant conflict of interest management agreement with the University of Pennsylvania Provost for Research. Research reported in this publication is solely the responsibility of the authors and does not necessarily represent the official views of the National Institutes of Health. L.E.P. also has equity in iFIT Prosthetics, LLC. All other authors have no other disclosures.
